# The Effect of Hydrogen Annealing on the Electronic Conductivity of Al-Doped Zinc Oxide Thin Films

**DOI:** 10.3390/ma18051032

**Published:** 2025-02-26

**Authors:** Ryoma Kawashige, Hideyuki Okumura

**Affiliations:** Graduate School of Energy Science, Kyoto University, Kyoto 606-8501, Japan; ammonite1030@gmail.com

**Keywords:** thin film, Al-doped ZnO, hydrogen annealing, optoelectronics, grain boundary scattering

## Abstract

In this research, Hall effect experiments and optical fittings were mainly conducted to elucidate the effect of hydrogen annealing on the electronic properties of polycrystalline Al-doped Zinc Oxide thin films by distinguishing the scattering by ion impurities and the scattering by grain boundaries. By comparing the carrier density and those mobilities of H_2_-annealed samples with Ar-annealed samples, the effect of H_2_ annealing was highlighted. AZO thin films were prepared on the quartz glass substrate at R.T. by an RF magnetron sputtering method, and the carrier density was controlled by changing the number of Al chips on the Zn target. After fabricating them, they were post-annealed in hydrogen or argon gas. Optical fitting was based on the Drude model using the experimental data of Near-Infrared spectroscopy, and the mobility at grain boundaries was analyzed by Seto’s theory. Other optical and crystalline properties were also checked by SEM, EDX, XRD and profilometer. It is indicated that the H_2_ annealing would improve both carrier density and mobility. The analysis referring to Seto’s theory implied that the improvement of mobility was caused by the carrier generation from introduced hydrogen atoms both at the grain boundary and its intragrain region. Furthermore, the effect of H_2_ annealing is relatively pronounced especially in low-doped region, which implies that Al and H have some interaction in AZO thin film. The interaction between Al and H in AZO thin film is still not confirmed, but this result implied that this interaction negatively affects the mobility at grain boundary.

## 1. Introduction

In the ongoing debate of energy transition towards cleaner production of energy, renewable energy, in particular solar energy, has become the most promising pathway. There have been several studies that have tried to optimize the cost of solar energy from analyzing the technology itself [1,2], to financial policy-based research [3,4,5] and even research that is focused on the soft costs and the impact of socioeconomics on the deployment of solar energy in various regions across the world [6,7,8]. This paper contributes to understanding the role of doping in thin films that can enable the increase in their conductivity, and eventually economic feasibility, to pave the pathway for utilization in smart building systems [1].

Zinc Oxide (ZnO) is a wide gap semiconductor with a band gap of 3.34 eV which is suitable for optoelectronic applications such as transparent electrodes because of its transparency in visible range light. Zinc is also abundant around the world and biocompatible with the human body. For these reasons, AZO (Aluminum-doped Zinc Oxide) thin film has been researched for many years as a promising alternative material for ITO (Indium-doped Tin Oxide). All these oxide thin films are collectively known as Transparent Conducting Oxides (TCOs). For advancing optoelectronics applications, TCOs are always expected to realize high electronic conductivity and transparency. At present, the conductivity of AZO has not surpassed that of ITO, which is exactly where this research is positioned.

In quite a few previous studies [9,10,11,12], it was reported that the AZO thin film acquires higher electronic conductivities by the introduction of hydrogen, including hydrogen annealing. Some researchers proposed that hydrogen atoms can work as a dopant in ZnO thin films by first-principle calculations [13,14,15]. But we have not accomplished the comprehensive understanding of the effect of hydrogen annealing. Therefore, in this study we conduct experiments to elucidate the mechanism of improving the electronic conductivity of AZO thin film by post-annealing in hydrogen gas.

The electronic conductivity σ of TCOs is described by Equation (1).

(1) σ=enμ

where e is the elementary charge, n is the carrier density and μ is the mobility of the carrier.

To improve the conductivity, both the carrier density, *n*, and the mobility *μ* have to be increased simultaneously. Increasing doping rate $n_{Al}$ can increase carrier density, but it can also increase ion impurity scattering to degrade the mobility, and increasing the carrier density can cause plasma absorption in the infrared region, which leads to a decrease in transmittance. So, improving the mobility without increasing the carrier density is the ideal way of achieving the desirable optoelectronic properties of TCOs.

T. Minami et al. [16] experimentally analyzed the mobility *μ* of polycrystalline AZO thin film and proved that the mobility is dominated by two factors: ionized impurity scattering in the highly doped region and grain boundary scattering in the low-doped region. From the results of [16], it can be postulated that the mobility in polycrystalline AZO thin film can be described as in Equation (2).

(2) $$\frac{1}{\mu }=\frac{1}{\mu_{ion}}+\frac{1}{\mu_{gb}}$$

where *μ*_ion_ corresponds to the mobility which only counts the ion impurity scatterings in the intra-grain regions and *μ*_gb_ corresponds to the mobility which only counts the scatterings at the grain boundaries. In this research, mainly two experiments were conducted to analyze the effect of H_2_ annealing, especially on the individual mobilities of *μ*_ion_ and *μ*_gb_:

- Hall effect experiment to measure the Hall mobility *μ*_Hall_ and the Hall carrier density *n*_Hall_.
- UV–Vis–NIR spectroscopy to calculate the optical mobility *μ*_opt_ from optical fitting based on the Drude model and to calculate the optical carrier density *n*_opt_. Using these, Equation (2) can be re-written as follows:
  (3) $$\frac{1}{\mu_{Hall}}=\frac{1}{\mu_{opt}}+\frac{1}{\mu_{gb}}$$

From Equation (3), we can express *μ*_gb_ as in Equation (4):

(4) $$\mu_{gb}=\frac{1}{\frac{1}{\mu_{Hall}}-\frac{1}{\mu_{opt}}}$$

As a result, *μ*_ion_ and *μ*_gb_ are known separately; wherein, each of the mobility terms can be explained by different theories: Brooks–Herring–Dingle theory for *μ*_ion_ [17] and Seto’s theory for *μ*_gb_ [18]. In Seto’s theory [18], mobility through the grain boundaries is derived as follows:

(5) $$\mu_{gb}=\mu_{0}exp(-\frac{\Phi_{B}}{kT})$$

$$\mu_{0}=\frac{eL}{\sqrt{2\pi m^{*}kT}}$$

where e is the elementary charge, L is the grain size and $m^{*}$ is the effective mass of free carrier, k is the Boltzmann constant, $\Phi_{B}$ is the potential barrier at grain boundaries, and T is the temperature.

In highly doped regions (*n*_Al_ > 2 at.%), $\Phi_{B}$ is derived as follows:

(6) $$\Phi_{B}=\frac{e^{2}Q_{t}^{2}}{8\varepsilon_{0}\epsilon_{r}n}$$

where *Q_t_* is the trap density at the grain boundary, $\varepsilon_{0}$ is the dielectric constant of free space, $\epsilon_{r}$ is the relative dielectric constant and n is the carrier density.

In Equation (6), *ϵ*_r_ = 8.12 [19], *n* = *n*_opt_ and *T* = 300 [*K*], which was assumed in this research. From Equations (5) and (6), *μ*_gb_ is dominated only by *Q_t_* and *n*.

## 2. Materials and Methods

One primary assumption in this study was to control the Al species. We adjusted the Al content in ZnO to change the carrier density, *n*, and also changed the gas species for post-annealing (Argon or Hydrogen) to assess how hydrogen affects the electronic properties of thin film by comparing the result with that of Argon gas. The purity of all gases was 99.9999%.

Polycrystalline AZO thin films were fabricated on the quartz glass substrate at room temperature with an RF magnetron sputtering machine (RSC-MG2, RIKEN, Wako, Japan). A pure Zinc target (purity: 99.9%) was set on the stage with tiny Al (Aluminum) chips on it. The distance between the target and the substrate was 15 cm and the RF power was set at 100 W. The gas flow rate in the chamber was set as Ar (argon) 30 sccm and O_2_ (oxygen) 30 sccm. Only the number of Aluminum chips were changed to control the Al content in the AZO thin film.

After making thin films with different Al contents, these samples were annealed in the quartz tube in the Argon or H_2_ (hydrogen) gas. The annealing temperature was 550 °C, and the annealing time was 30 min. After putting the quartz tube with the samples in the furnace at 550 °C, the temperature went down once then came back to 550 °C after a while, and 30 min was measured from coming back to 550 °C. The gas pressure in the quartz tube was set at 1 atm (at 550 °C) for all samples. Only the gas species (Argon or Hydrogen) was changed to simply compare these results.

In addition to these experiments, some experiments such as X-ray diffraction were conducted to evaluate the crystal structures, and SEM and EDX were conducted to mainly evaluate the elemental composition. After post-annealing, the physical properties were checked by XRD, SEM, EDX and profilometer to measure the film thickness. Then, finally, these samples were analyzed by the Hall effect experiment and UV–Vis–NIR spectroscopy.

### 2.1. X-Ray Diffraction (XRD)

**Figure 1:**
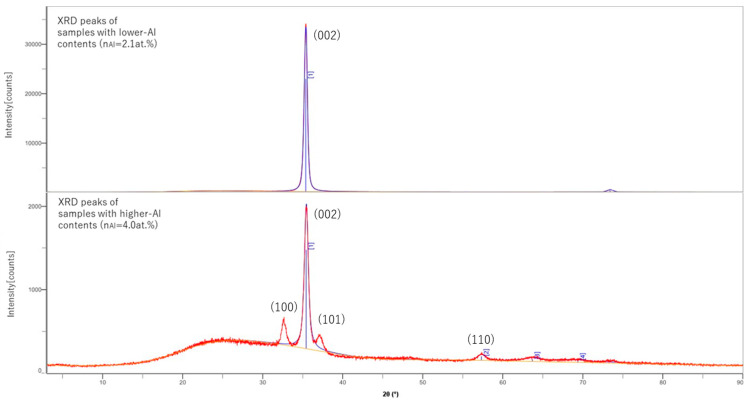
XRD peaks of samples with different Al content.

RINT2100CMJ (RIGAKU, Akishima, Japan) was used to conduct out of plane XRD analysis with Cu-Kα X-ray. It was shown that all the samples have a peak at roughly 2θ = 35.2°, which corresponds to the (002) orientation. However, at highly doped regions ($n_{Al}$ > 4.0 at.%), other peaks started to appear at roughly 2θ = 32.5°, 37.0° and 57.1°, and other tiny peaks appeared in high-angle regions, as shown in Figure 1. These tiny peak positions could not be precisely specified. These peaks were all corresponding to other ZnO orientation peaks, like (100) for 32.5°, (101) for 37.0° and (110) for 57.1°. This shows that (002) was prioritized in the low-doped region, but as the Al-doping rate increased, the growth of (002) was hindered by Al-related impurities and other peaks started to appear.

These peak positions are slightly different from the literature data mainly because of the peak shift by Al dopant replacing Zn atoms. The lattice constants were changed for the Al-Zn replacement. In this experiment, the (002) peak position of pure ZnO thin film was also detected at 35.2° (literature data: 2θ = 34.4° [9]), which can be explained by the fact that ZnO crystal structure shrinks from the default because of the spontaneous polarization of ZnO in the direction of (002).

**Figure 2:**
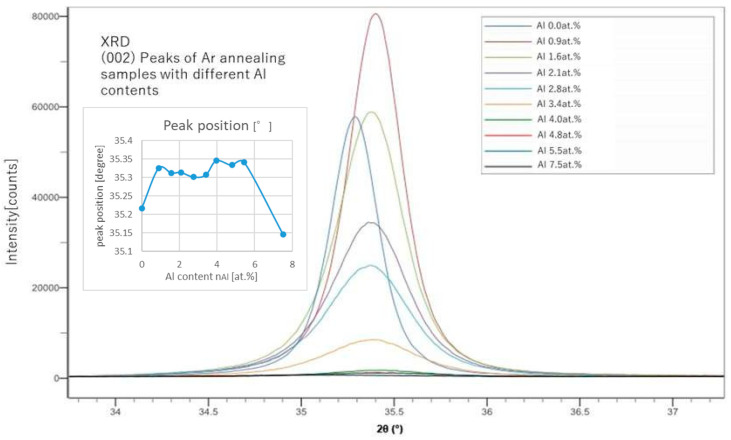
XRD (002) peaks of samples with different Al contents (after Ar annealing).

**Figure 3:**
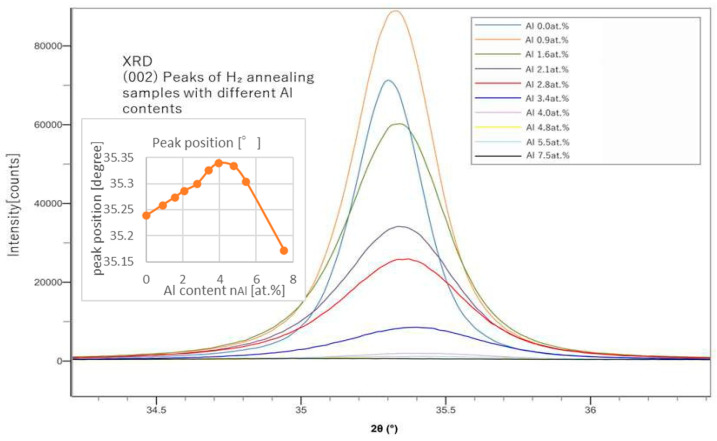
XRD (002) peaks of samples with different Al contents (after H_2_ annealing).

From the peak positions of (002), we can calculate the lattice constant of the c-axis and estimate the replacing rate of Al with Zn. From the peak shapes of (002) shown in Figure 2 and Figure 3, we also evaluated the grain size, *L*, using the Sherrer’s equation (Equation (7)).

(7) $$L=\frac{K\lambda }{Bcos\theta }$$

Here, *K* = 0.89, θ is the peak position, λ is the wavelength of Cu-Kα X-ray (=0.15418 nm) and *B* is the full width at half maximum (FWHM).

**Figure 4:**
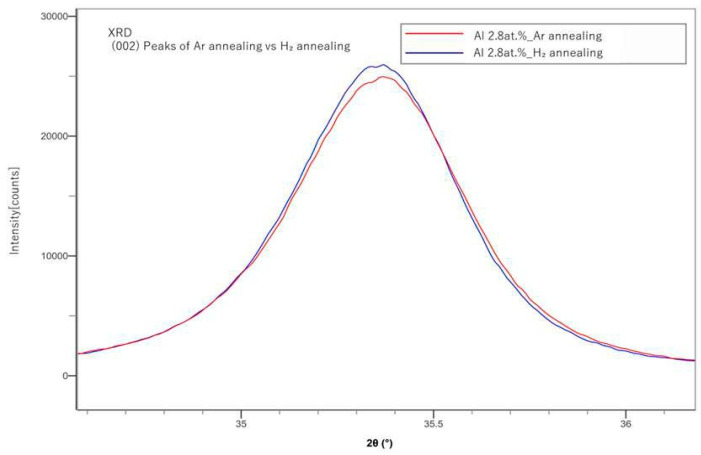
Comparison of the XRD (002) peaks of Ar and H_2_-annealed samples.

In Figure 4, (002) peaks of the Ar-annealed samples and the H_2_-annealed samples were compared. H_2_ annealing showed slightly sharper peaks than those of the Ar-annealed samples. In Figure 1, Figure 2, Figure 3 and Figure 4, instrumental broadening was corrected with the crystalline material of LaB6, which is an established standard in XRD measurements. The peaks for all cases mostly appeared between 35 and 35.5 degrees, which is in line with the results presented by a previous study [20].

### 2.2. Scanning Electron Microscope (SEM)

An SU6600 (HITACHI High-Tech Corp., Minato, Japan) was used to observe the film surface and the cross section of the film. The acceleration voltage was set at 20 kV. The film surface was coated via gold sputtering prior to SEM observation to prevent charging of samples, and the surface geometry and the film thickness were observed.

### 2.3. Energy Dispersive X-Ray Spectroscopy (EDX)

The composition ratio of Zn:O:Al was evaluated with the EDX (Bruker, Billerica, MA, USA) attached to the SEM.

### 2.4. Profilometer

The film thickness was directly measured by the profilometer DEKTAC150 (ULVAC, Chigasaki, Japan).

### 2.5. Hall Effect Experiment

A ResiTest8300 (TOYO Corp., Tokyo, Japan) was used with a 0.67 T magnet in the van de Pauw method. The samples were cut into 10 × 10 mm, and the substrate and the pins were directly attached to the corners of the squared samples.

### 2.6. UV–Vis–NIR Spectroscopy

Transmittance in Ultraviolet, Visible and Near-Infrared ranges (wavelength from 300 nm to 2500 nm through the thin films) ware measured with Lambda750S (PerkinElmer, Waltham, MA, USA). From the transmittance in the range of Ultraviolet light (wavelength from 300 nm to 380 nm), we estimated the band gap *E*_g_ of the thin film using a Tauc plot. As the doping rate *n*_Al_ increased, *E*_g_ also increased. This can be explained by Burstein–Moss effect described in Equation (8).

(8) $$\Delta E_{g}=E_{g}-E_{g0}=\frac{\hbar^{2}}{{2m}_{vc}^{*}}{\left(3\pi^{2}n_{opt}\right)}^{\frac{2}{3}}$$

where *E*_g_ is the band gap obtained from the Tauc plot, *E*_g0_ is the band gap of non-doped ZnO, which was 3.22 eV in this research, and *m*^∗^ is the reduced effective mass of the free carrier, which was 0.69 *m*_0_ (*m*_0_ is the mass of electron of free space) in this research. N_opt_ is the optical carrier density.

By using the transmittance in the range of Near-Infrared light (wavelength from 1000 nm to 2500 nm), the optical fitting was calculated to estimate the optical mobility *n*_opt_ based on the Drude model. The procedure of optical fitting is described below:

In the Drude model, the dielectric function *ε* = *ε*_l_ + *ε*_2_ is described in Equations (9) and (10) [21].

(9) $$\varepsilon_{1}=\varepsilon_{\infty }-\frac{ne^{2}}{m^{*}\varepsilon_{0}}\frac{1}{\omega^{2}+\gamma^{2}}$$

(10) $$\varepsilon_{2}=\frac{ne^{2}}{m^{*}\varepsilon_{0}}\frac{\gamma }{\omega^{2}+\gamma^{2}}$$

where *ε*_l_ and *ε*_2_ are real and imaginary parts of the dielectric function, *n* is the carrier density, *m*^∗^ is the effective mass of the free carrier, *e* is the elementary charge, $\varepsilon_{0}$ is the dielectric function of free space, $\varepsilon_{\infty }$ is the dielectric constant of the background, *ω* is the frequency of the electronic magnetic wave and γ is the damping constant. For the parameters used in the Drude model, the assumptions of Equations (11) and (12) were applied.

(11) $$n=n_{Hall}$$

(12) $$m^{*}=m_{0}^{*}\sqrt{1+2C\frac{\hbar^{2}}{m_{0}^{*}}{\left(3\pi^{2}n_{opt}\right)}^{\frac{2}{3}}}$$

where *m*^∗^ is the effective mass of free carrier at the bottom of the conduction band in ZnO. In this research, *m*^∗^ was taken as follows: *m*^∗^ = 0.28 *m*, where *m* is the electron mass of free space. For *m*^∗^, the non-parabolicity of the conduction band of ZnO was assumed [22].

Refractive index *n*_r_ and extinction coefficient κ are described in Equations (13) and (14).

(13) $$n_{r}=\sqrt{\frac{\sqrt{\varepsilon_{1}^{2}+\varepsilon_{2}^{2}}{+\varepsilon }_{1}}{{2\varepsilon }_{0}}}$$

(14) $$\kappa =\sqrt{\frac{\sqrt{\varepsilon_{1}^{2}+\varepsilon_{2}^{2}}{-\varepsilon }_{1}}{{2\varepsilon }_{0}}}$$

Transmittance, *T*, is derived from the law of reflection and Lambert–Beer’s law (Equation (15)).

(15) $$T=\left(1-R\right)e^{-\alpha d}=\left\{1-\frac{{{(n}_{r}-1)}^{2}+\kappa }{{{(n}_{r}+1)}^{2}+\kappa }\right\}e^{-\frac{2}{c_{0}}\kappa \omega d}$$

*R* is the reflectance, *α* is the absorption coefficient and d is the film thickness given from the profilometer.

From Equations (9), (10), (13), (14) and (15), the transmittance (*ω*) could be expressed only by two changeable parameters, $\varepsilon_{\infty }$ and *γ*. (All other constants were taken from other experiments.) The fitting parameters $\varepsilon_{\infty }$ and *γ* were determined by the least squares method, and the theoretical transmittance was fitted to the transmittance spectrum obtained from NIR spectroscopy. Here, *γ* is the damping constant, and *μ*_opt_ is related to *γ* as shown in Equation (16).

(16) $$\mu_{opt}=\frac{e}{m^{*}\gamma }$$

From this relation, the optical carrier mobility $\mu_{opt}$ was finally obtained.

## 3. Results and Discussions

### 3.1. Crystalline Properties

**Figure 5:**
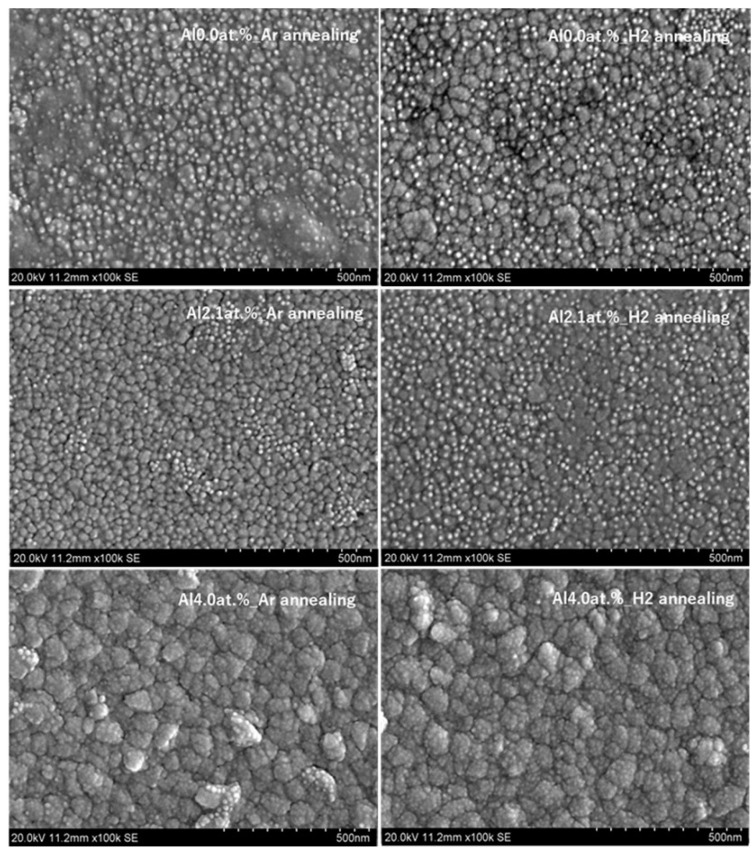
SEM images of crystalline surfaces under different conditions and annealing gases.

**Figure 6:**
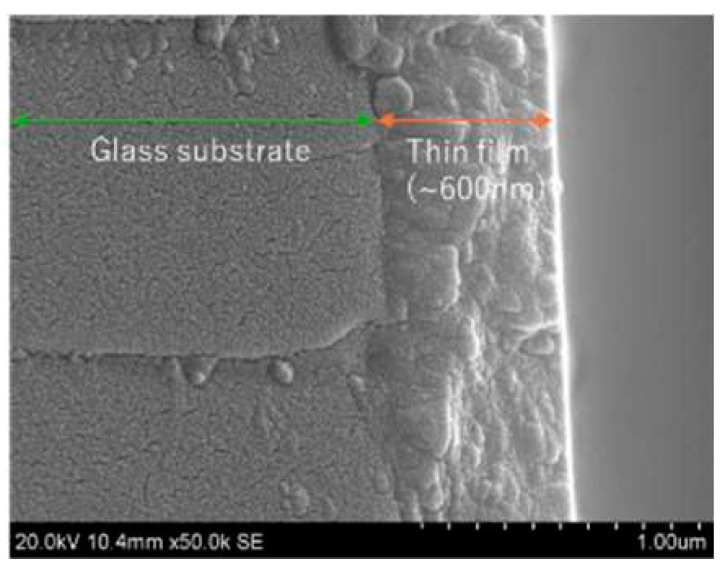
Cross section of the thin film as seen through the SEM ($n_{Al}$ = 2.1 at.%). The white parts indicate a larger amount of free electrons. The quartz glass was cleaned by ultrasonic washing machine in acetone before the formation of AZO films.

Figure 5 is one of the SEM pictures of a surface with different Al contents and annealing gases. The image shows that the surface of thin film is composed of numerous grains. The film thickness is around 600 nm as seen in Figure 6. In both Figure 5 and Figure 6, the working distance was 7 mm to optimize the measurements accordingly for a definitive resolution.

**Figure 7:**
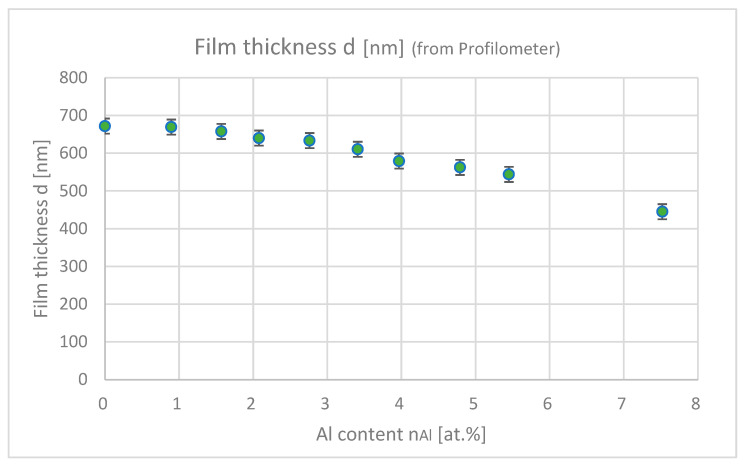
Film thicknesses of the samples with differing Al contents (each plot shows the average film thickness for an Ar-annealing sample and a H_2_-annealing sample).

Figure 7 shows the film thicknesses of samples with differing Al contents. As the Al content increases, the film thickness decreases because of Al-induced impurities, which inhibits the film growth.

**Figure 8:**
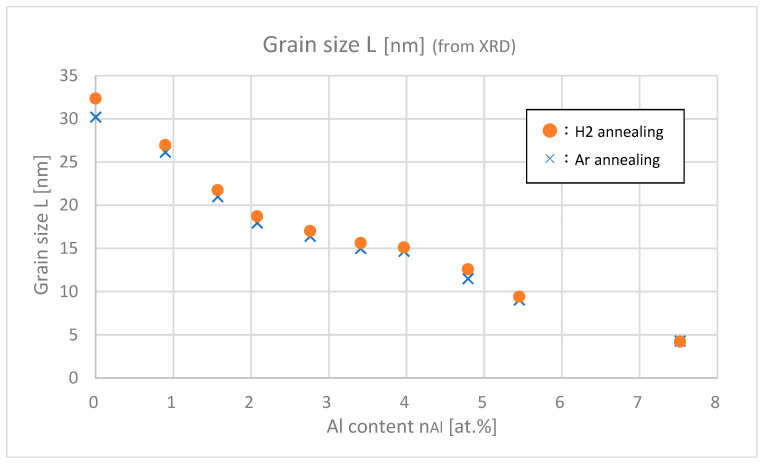
Grain sizes of the samples with differing Al contents.

Figure 8 shows the estimated grain sizes of samples with differing Al contents. As for both argon and hydrogen annealing, the grain size decreases as Al content increases. The decrease in film thickness shown in Figure 7 is consistent with this decrease in grain size. Furthermore, if we focus on details, the samples with H_2_ annealing have slightly (about 8 Å) larger grain sizes than the samples with Ar annealing. This implies that H_2_ annealing can support the growth of grains much more efficiently.

**Figure 9:**
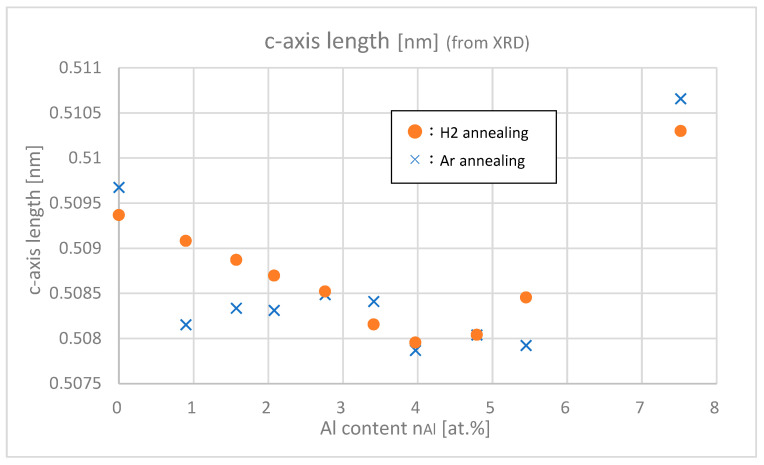
**c**-axis lengths of the samples with differing Al contents.

Figure 9 shows the c-axis lengths of samples with different Al contents. For both Ar and H_2_ annealing, c-axis length decreased until Al content reached around 4.0 at.% and then increased above that value. According to Vegard’s law, the c-axis length is predicted to proportionally decrease as Al content increases because of the smaller atomic size ratio of Al atom compared with Zn atom. The c-axis length, however, increased in *n*_Al_ > 4.0 at.% range, which implies that Al interstitial impurity increased in highly doped regions and caused the expansion of the lattice. This means that it becomes less helpful to add Al at *n*_Al_ > 4.0 at.% to get higher carrier densities. (This is one of the motivations for co-doping hydrogen to Al.)

### 3.2. Optical and Electronic Properties

**Figure 10:**
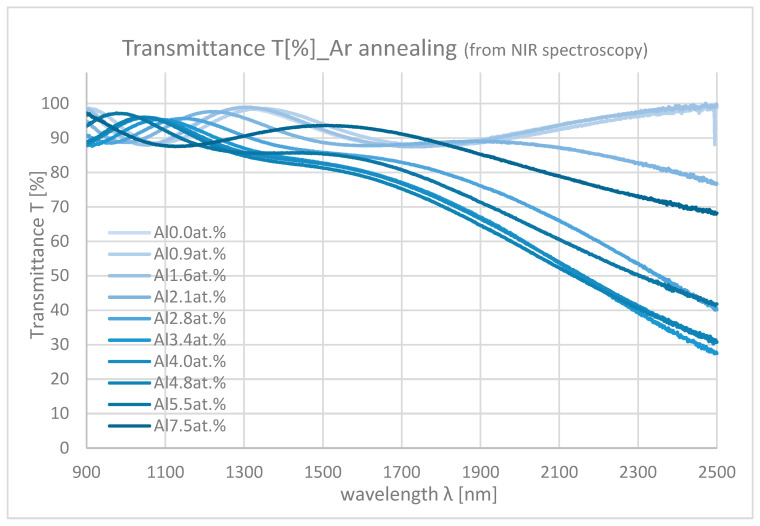
Transmittances of the samples with differing Al contents in NIR range (Ar annealing).

**Figure 11:**
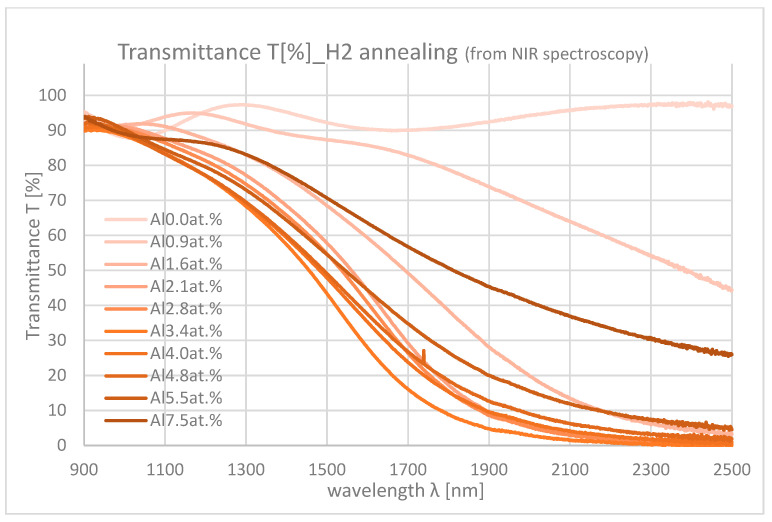
Transmittances of the samples with differing Al contents in NIR range (H_2_ annealing).

Figure 10 and Figure 11 show the NIR spectroscopy of samples with differing Al contents. In the NIR range, transmittance decreases from about 90% to 0% mainly due to the absorption by free carriers. As the Al content increases, the absorption also increases because of the increase in carrier density.

**Figure 12:**
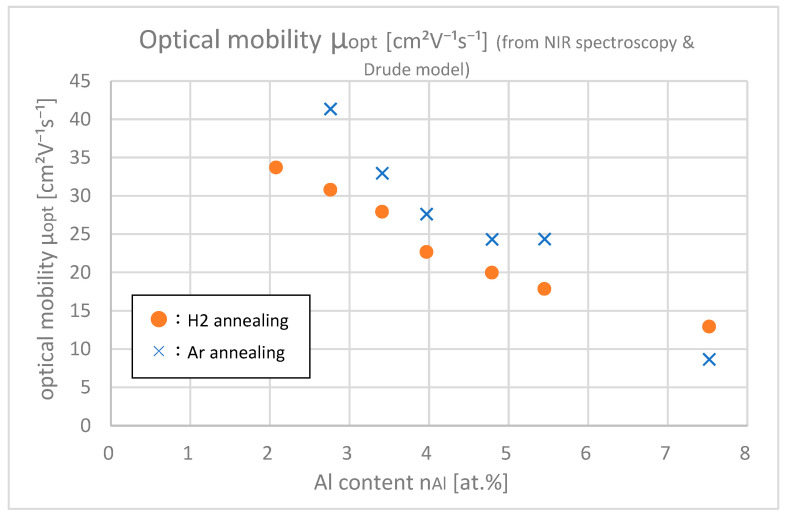
Optical mobilities *μ*_opt_ of the samples with differing Al contents.

Figure 12 shows the optical mobilities $\mu_{opt}$ of samples with different Al contents obtained from optical fitting. Only in the range that the absorption was clearly found ($n_{Al}$ > 2.5 at.%) was optical fitting possible. This proves that samples with Ar annealing have larger optical mobilities than samples with H_2_ annealing. Comparison of Ar annealing with H_2_ annealing shows that the ion impurity scattering in the intragrain region increased after H_2_ annealing compared to Ar annealing. This can be explained by the process where hydrogen gets introduced in the bulk via H_2_ annealing, which essentially works as a dopant, and H^+^ increased the ion impurity scattering.

**Figure 13:**
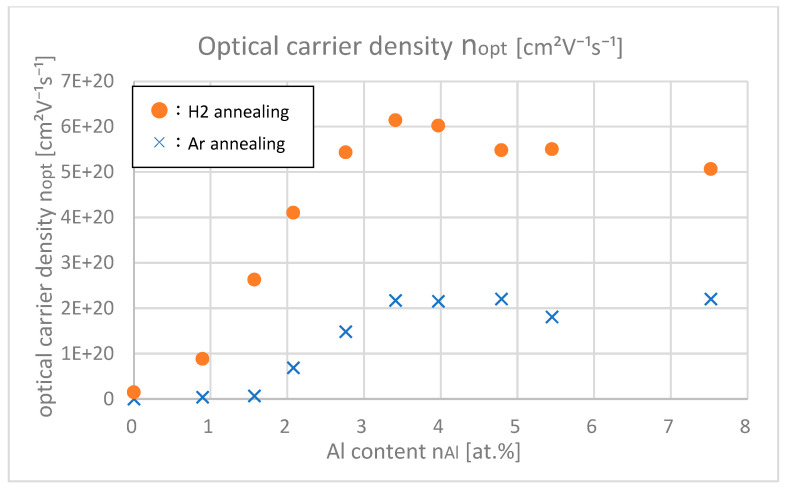
Optical carrier densities of the samples with differing Al contents.

**Figure 14:**
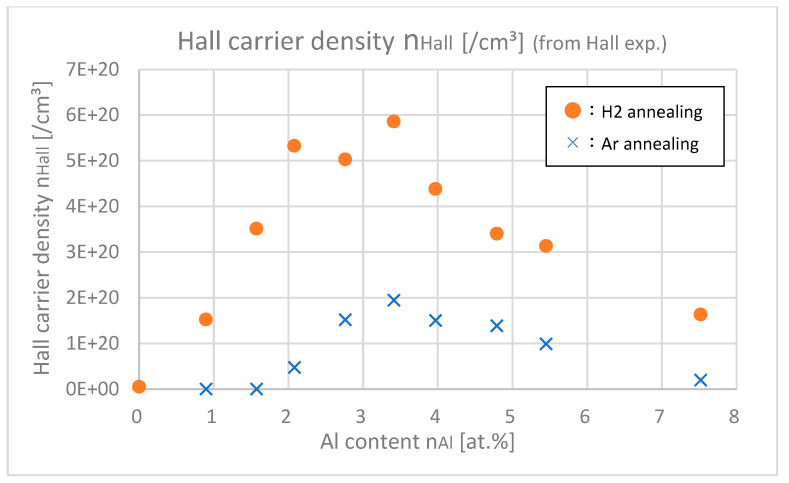
Hall carrier densities of the samples with differing Al contents.

Figure 13 shows the optical carrier densities $\mu_{opt}$ of samples with different Al contents, which were calculated from the Tauc plot in the UV light range. Figure 14 shows the Hall carrier densities *n*_Hall_ of samples with different Al contents.

From Figure 13 and Figure 14, it was found out that *n*_opt_ are larger than *n*_Hall_ especially in highly doped ranges (*n*_Al_ > 3.5 at.%). In the highly doped range, *n*_opt_ was almost saturated, but *n*_Hall_ decreased. This is probably because the gross amount of free carrier (=*n*_opt_) created by Al or H is saturated after doping about 4.0 at.% of Al, but the net amount of free carrier that remained in the intragrain regions decreased due to the increase in trapped carriers at the grain boundaries. As shown in Figure 8, as the Al content increased, the grain size decreased; that is, the total amount of grain boundaries in the thin film increased, which led to the decrease in net free carrier in the intragrain regions.

**Figure 15:**
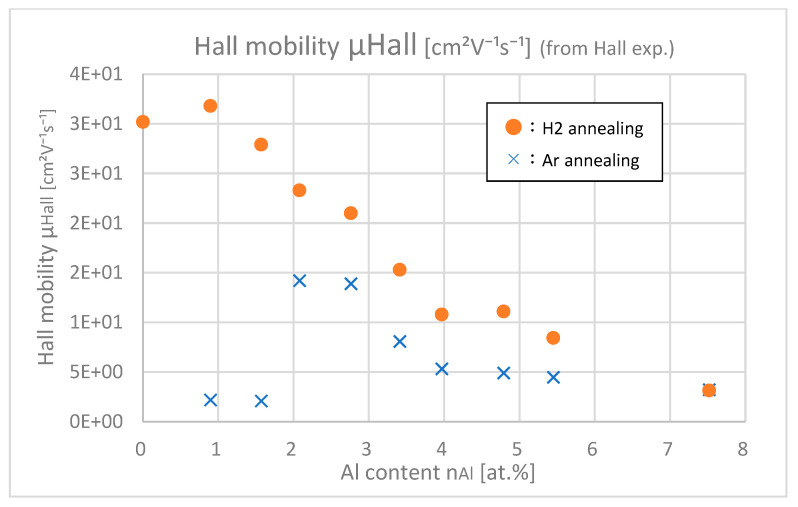
Hall mobilities of the samples with differing Al contents.

Figure 15 shows the Hall mobilities of samples with different Al contents. At any Al content, especially in low-doped samples, Hall mobilities of H_2_-annealing samples are larger than those of Ar-annealing samples. As the Al content increases, this difference decreases. This result means that the Al–H interaction negatively affects the improvement of *μ*_Hall._

**Figure 16:**
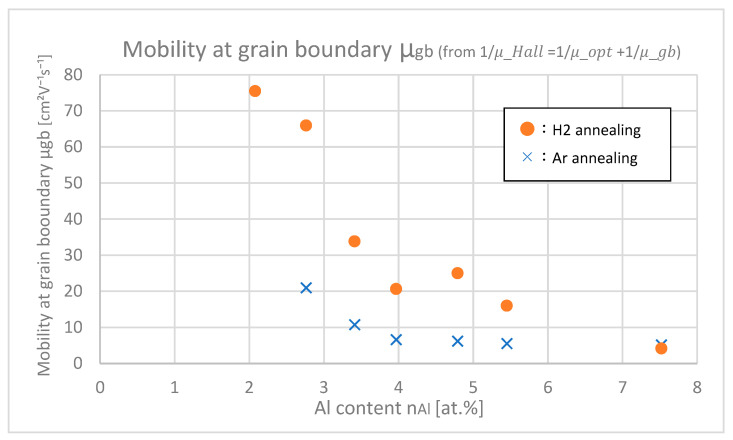
Mobility at the grain boundaries of the samples with differing Al contents.

From Figure 12 and Figure 15 and Equation (4), the mobilities at grain boundaries *μ*_gb_ were calculated, which is shown in Figure 16. At any Al contents, *μ*_gb_ of H_2_-annealed samples are larger than those of Ar-annealed samples. This shows that H_2_ annealing improves mobility at the grain boundaries.

**Figure 17:**
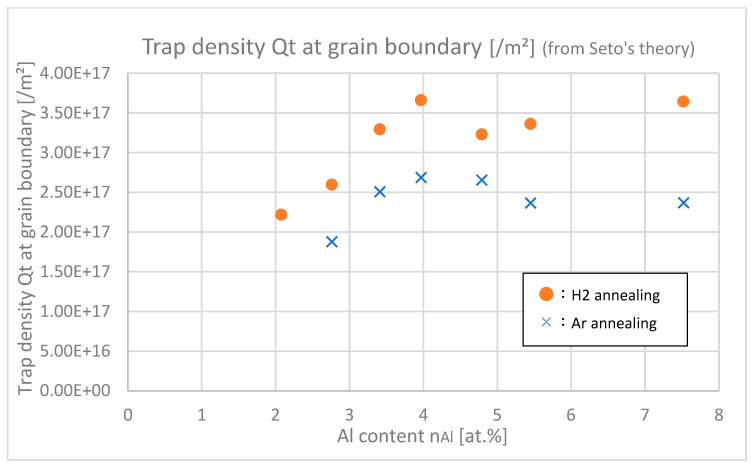
Trap densities *Q*_t_ of the samples with differing Al contents.

In Figure 17, the trap densities *Q*_t_ at grain boundaries of samples with differing Al contents are shown. Trap densities of H_2_-annealed samples at any Al contents were larger than those of Ar-annealed samples, which indicates that H_2_ annealing increases the trap densities. This is due to the fact that hydrogen itself or its complex works as a trap at grain boundaries. The increase in trap density negatively affects *μ*_gb_; nevertheless, H _2_ annealing finally increased *μ*_gb_ as shown in Figure 16. This is probably because carrier density *n* increased after H_2_ annealing as shown in Figure 13 and Figure 14, and then subsequently the potential barriers at grain boundaries decreased, which was relatively dominant that the *μ*_gb_ was eventually increased by H_2_ annealing.

## 4. Conclusions

In summary, the effects of H_2_ annealing on the electronic properties of AZO thin film were concluded as follows (all results are from comparisons with Ar annealing):

- H_2_ annealing increases the carrier density and it decreases the potential barriers at grain boundaries. (The mechanism by which hydrogen acts as a dopant has been proposed by several first-principles studies [13,14,15].)
- The effect of H_2_ annealing is relatively influential, especially in low-doped regions, which implies that Al and H have some interaction in AZO thin film.
- The interaction between Al and H in AZO thin film is still not verified, but these results implied that the interactions negatively affect the mobility at grain boundaries.

To clarify, the originality of this paper lies in the combined application of optical fitting, the premise of separating the carriers into two parts (carriers in the bulk and carriers in the grain boundary) and Seto’s theory to the AZO + H thin film system. These ideas have been widely used, but we recognize that the combined application of these ideas to an AZO + H thin film system with the results of experiments have not been reported so far.

It is hoped that these conclusions will be verified by more direct measurements, such as measuring the potential barrier height at grain boundaries or directly observing hydrogen in the sample. It is also hoped that the mechanism by which hydrogen acts as a dopant and improves mobility will be elucidated by more microscopic investigations such as first principles calculations or defect level measurements.

## Data Availability

The original contributions presented in this study are included in the article. Further inquiries can be directed to the corresponding author.

## References

[1] Asim N., Sopian K., Ahmadi S., Saeedfar K., Alghoul M.A., Saadatian O., Zaidi S.H. (2012). A review on the role of materials science in solar cells. Renew. Sustain. Energy Rev..

[2] Kennedy R. (2016). Solar LCOE Now 29% Lower than Any Fossil Fuel Option, Says EY. PV Magazine.

[3] Gao X., Liu J., Zhang J., Yan J., Bao S., Xu H., Qin T. (2013). Feasibility evaluation of solar photovoltaic pumping irrigation system based on analysis of dynamic variation of groundwater table. Appl. Energy.

[4] Frysztacki M., Brown T. (2020). Modeling Curtailment in Germany: How Spatial Resolution Impacts Line Congestion. Proceedings of the 2020 17th International Conference on the European Energy Market (EEM).

[5] Basu S., Ishihara K.N. (2023). Multivariate time–frequency interactions of renewable and non-renewable energy markets with macroeconomic factors in India. Energy Syst..

[6] Dumlao S.M.G., Ishihara K.N. (2020). Reproducing solar curtailment with Fourier analysis using Japan dataset. Energy Rep..

[7] Basu S., Ogawa T., Okumura H., Ishihara K.N. (2021). Assessing the geospatial nature of location-dependent costs in installation of solar photovoltaic plants. Energy Rep..

[8] Basu S., Hoshino T., Okumura H. (2024). Analyzing Geospatial Cost Variability of Hybrid Solar–Gravity Storage System in High-Curtailment Suburban Areas. Energies.

[9] Jia J., Oka N., Kusayanagi M., Nakatomi S., Shigesato Y. (2014). Origin of carrier scattering in polycrystalline Al-doped ZnO films. Appl. Phys. Express.

[10] Park Y.R., Kim J., Kim Y.S. (2009). Effect of hydrogen doping in ZnO thin films by pulsed DC magnetron sputtering. Appl. Surf. Sci..

[11] Zhu B.L., Wang J., Zhu S.J., Wu J., Zeng D.W., Xie C.S. (2012). Optimization of sputtering parameters for deposition of Al-doped ZnO films by rf magnetron sputtering in Ar+H_2_ ambient at room temperature. Thin Solid Film..

[12] Yamada T., Makino H., Yamamoto N., Yamamoto T. (2010). Ingrain and grain boundary scattering effects on electron mobility of transparent conducting polycrystalline Ga-doped ZnO films. J. Appl. Phys..

[13] Oba F., Choi M., Togo A., Tanaka I. (2011). Point defects in ZnO: An approach from first principles. Sci. Technol. Adv. Mater..

[14] Janotti A., Van de Walle C.G. (2007). Native point defects in ZnO. Phys. Rev. B.

[15] Li L., Zhang Z., Wang J., Yang P. (2022). Improving photoelectric perfomance with hydrogen on Al-doped ZnO. Mater. Chem. Phys..

[16] Minami T., Miyata T., Tokunaga H. (2019). Electron Scattering from Disordered Grain Boundaries in Degenerate Polycrystalline Al-Doped ZnO Thin Films. Phys. Status Solidi.

[17] Brooks H., Herring C. (1951). Scattering by Ionized Impuri-ties in Semiconductors. Phys. Rev..

[18] Seto J.Y.W. (1975). The electrical properties of polycrystalline silicon films. J. Appl. Phys..

[19] Look D.C., Leedy K.D., Tomich D.H., Bayraktaroglu B. (2010). Mobility analysis of highly conducting thin films: Application to ZnO. Appl. Phys. Lett..

[20] Miccoli I., Spampinato R., Marzo F., Prete P., Lovergine N. (2014). DC-magnetron sputtering of ZnO:Al films on (00.1)Al_2_O_3_ substrates from slip-casting sintered ceramic targets. Appl. Surf. Sci..

[21] Dingle R.B. (1955). XCIV. Scattering of electrons and holes by charged donors and acceptors in semiconductors. Lond. Edinb. Dublin Philos. Mag. J. Sci..

[22] Pisarkiewicz T., Zakrzewska K., Leja E. (1989). Scattering of charge carriers in transparent and conducting thin oxide films with a non-parabolic conduction band. Thin Solid Film..

